# Avaliação da Circulação Coronariana após a Cirurgia de Jatene

**DOI:** 10.36660/abc.20200095

**Published:** 2021-06-08

**Authors:** Mariana Nicoletti Ferreira Baldo, Henrique Simão Trad, Tarcisio José da Silva, Paulo Henrique Manso

**Affiliations:** 1 Universidade de São Paulo Ribeirão PretoSP Brasil Universidade de São Paulo , Ribeirão Preto , SP - Brasil; 2 Lotus Radiologia Ribeirão PretoSP Brasil Lotus Radiologia , Ribeirão Preto , SP – Brasil

**Keywords:** Artéria Coronária, Cirurgia de Jatene Transposição das Grandes Artérias, Circulação Coronária, Tomografia Computadorizada por Raios X, Diagnóstico por Imagem

## Abstract

**Fundamento:**

A avaliação da artéria coronária após a cirurgia de Jatene ainda é um desafio clínico.

**Objetivo:**

Correlacionar alterações anatômicas identificadas por tomografia computadorizada cardíaca (TCC) com alterações fisiológicas detectadas na avaliação clínica para diagnosticar obstrução coronária no pós-operatório tardio de pacientes submetidos à cirurgia de Jatene.

**Métodos:**

Este estudo incluiu 61 pacientes consecutivos com idade média de 9,4 anos que foram submetidos à cirurgia de Jatene. Os pacientes realizaram ecocardiografia, eletrocardiografia, teste cardiopulmonar do exercício, e tomografia computadorizada cardíaca para avaliação da capacidade funcional e anatomia da artéria coronária.

**Resultados:**

A tomografia computadorizada cardíaca revelou que somente 3,3% dos pacientes apresentaram estenose da artéria coronária. Esses pacientes eram assintomáticos, e não foram detectados sinais de isquemia miocárdicas pelos exames realizados.

**Conclusão:**

A incidência de anormalidades da artéria coronária é de 3,3% no seguimento tardio de nossa coorte de pacientes submetidos à cirurgia de Jatene. Não existe uma diretriz clara sobre o porquê, quando, e como esses pacientes deveriam ser rastreados, ou o que propor quando pacientes assintomáticos forem diagnosticados com obstrução coronária.

## Introdução

Apesar de a cirurgia de transposição de grandes vasos (cirurgia de Jatene) estar associada com baixa taxa de mortalidade precoce e baixa morbidade na maioria dos centros, ^1^ complicações tardias, tais como lesões obstrutivas da artéria coronária, obstrução da via de saída do ventrículo esquerdo, regurgitação e dilatação da valva neoaórtica podem estar presentes em até 25% dos casos. ^2^ Além disso, a ocorrência de anormalidades tardias na circulação da artéria coronária têm sido relatadas em até 18% dos casos. ^3^ No entanto, a real incidência de problemas tardios da artéria coronária após a operação de Jatene é desconhecida, uma vez que a maioria dos pacientes que apresentam estenose ou oclusão da artéria coronária podem ser assintomáticos, e as incidências relatadas tendem a depender da profundidade das investigações. ^4^ Ainda, não existem diretrizes claras sobre quando esses pacientes deveriam ser rastreados ou sobre o melhor método de rastreamento nessa situação. ^5^

Além da avaliação clínica, pacientes submetidos à cirurgia de Jatene requerem uma avaliação multimodal de possíveis complicações tardias. A ecocardiografia transtorácica (ETT), a eletrocardiografia (ECG), e o teste cardiopulmonar de exercício (TCPE) são normalmente utilizados no acompanhamento em longo prazo desses pacientes. Contudo, esses métodos de rastreamento não são suficientemente sensíveis para detectar anormalidades na artéria coronária. ^6^

A tomografia computadorizada cardíaca (TCC) é um bom método de avaliação da anatomia da artéria coronária no pós-operatório tardio de pacientes submetidos à cirurgia de Jatene, com uma elevada resolução espacial em um curto tempo de aquisição. A TCC é um método ideal para pacientes que necessitam de uma avaliação detalhada das artérias coronárias reimplantadas. ^7^

## Objetivo

Avaliar a circulação da artéria coronária no pós-operatório tardio em uma coorte de pacientes submetidos à cirurgia de Jatene utilizando ECG, ETT, TCPE, e TCC.

## Métodos

Este estudo prospectivo foi aprovado pelo comitê de ética local (43493315.6.0000.5440). Foram recrutados pacientes submetidos à cirurgia de Jatene em nossa instituição entre 1998 e 2009. Critérios de inclusão foram idade maior que cinco anos e consentimento por escrito dos pais para participação no estudo. Pacientes alérgicos a contraste iodado foram excluídos do estudo. Dados clínicos relacionados ao diagnóstico inicial, anatomia da artéria coronária, e idade na cirurgia foram coletados dos prontuários médicos. Os pacientes foram então submetidos à ETT, ECG, TCC, e TCPE em um período de quatro meses.

A ECG e a ETT foram realizadas de acordo com protocolos de rotina. O TCPE foi conduzido em uma esteira inclinada, com um aumento na inclinação durante o teste.

A TCC foi realizada em um aparelho de 64 cortes (Somaton Sensation, Siemens, Alemanha). Quando necessário, um betabloqueador oral foi administrado duas horas antes do exame, para atingir uma frequência cardíaca menor que 80 bpm.

As crianças participantes foram treinadas anteriormente para segurar o fôlego por 10 segundos. Quando o paciente não era capaz de cooperar, foi administrada midazolam endovenosa (0,1 a 0,2 mg/Kg). Parâmetros de aquisição de imagem foram adaptados para se utilizar a dose mais baixa possível de radiação. Dois radiologistas independentes analisaram as imagens.

### Análise estatística

Foi realizada uma análise descritiva dos dados. Os dados contínuos foram expressos em mediana e intervalos, e os dados categóricos expressos como porcentagem. Todas as análises foram realizadas usando o programa GraphPad Prism 5.0 (GraphPad Software, La Jolla, CA, EUA), com o nível de significância estatística definido como p< 0,05.

## Resultados

### Características demográficas dos pacientes

Dos 69 pacientes inicialmente recrutados, quatro não concordaram em participar, dois perderam seguimento, e dois morreram (um durante o implante de *stent* na artéria pulmonar no laboratório de cateterismo, e um paciente morreu por causa desconhecida). Os 61 pacientes restantes foram submetidos à ECG e ETT; 60 pacientes foram submetidos à TCC; e 52 pacientes realizaram o TCPE. Todos os pacientes eram assintomáticos e não usavam nenhuma medicação para doença cardiovascular.

Setenta porcento dos pacientes eram do sexo masculino. A maioria dos pacientes apresentaram transposição das grandes artérias (TGA) com septo interventricular intacto (56,7%); 31,7% apresentaram TGA com defeito do septo ventricular (DSV); e 11% apresentaram anomalia de Taussig-Bing. No pré-operatório, a origem da artéria coronária esquerda e da artéria coronária direita foi normal em 90% dos pacientes (seio 1 e 2, respectivamente).

### Abordagem cirúrgica

A cirurgia de Jatene foi realizada em um período mediano de 14 dias (2 a 38 dias); a idade mediana dos participantes foi 9,4 anos (5 a 18 anos), o peso mediano foi 29,9 Kg (20 a 84 Kg); e a altura mediana 134 cm (112 a 183 cm).

### Investigação clínica

Todos os pacientes possuíam resultados de ECG e ETT. A maioria (96%) estava em ritmo sinusal, e 4% em ritmo atrial direito. Nenhum paciente apresentou alterações no segmento ST ou ectopia ventricular no ECG de repouso. A ETT mostrou que todos os pacientes apresentaram fração de ejeção normal (>55%) e motilidade regional da parede ventricular normal.

TCPE: Cinquenta e um pacientes conseguiram realizar o TCPE (10 pacientes apresentavam condições neurológicas ou em algum membro que os impedia de realizar o teste), e nenhum apresentou anormalidades no seguimento ST ou arritmia durante o teste. Os pacientes apresentaram um VO _2_ máximo de 31,7 mL/Kg/min (22,3 – 43,2) e atingiram uma média de 9 (6,4-12,3) METS.

**Figura 1:**
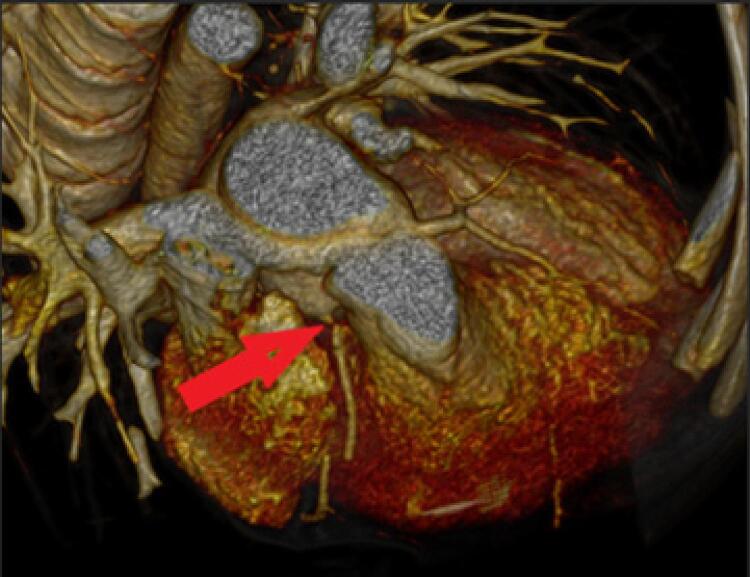
Imagem da tomografia computadorizada cardíaca; artéria coronária direita com obstrução grave (flecha).

TCC: Sessenta pacientes realizaram TCC. O produto dose-comprimento (DLP, *dose length product* ) médio foi 138 (56 -490) mGy-cm; a dose média foi 2 (0,9–8,7) mSv. Somente dois pacientes (3.3%) apresentaram anormalidades na artéria coronária; um apresentou estenose moderada da artéria coronária esquerda, e o outro obstrução grave da artéria coronária direita ( Figura 1 ). Nós classificamos o grau de obstrução da artéria coronária de acordo com diretrizes pulicadas. ^8^ Ambos os pacientes eram assintomáticos, e os resultados em todos os outros testes (ECG de repouso, ETT e TCPE) eram normais ( Tabela 1 ). Não encontramos nenhuma correlação entre o diagnóstico cardíaco primário ou padrão da artéria coronária e a presença de estenose coronária.

**Tabela 1:** – Características dos pacientes com anormalidades da artéria coronária segundo tomografia computadorizada cardíaca (TCC)

	Paciente 1	Paciente 2
Lesão da artéria coronária	Oclusão moderada da artéria coronária esquerda	Obstrução grave da artéria coronária direita
Idade (anos)	14,7	6,6
Sexo	Feminino	Masculino
Diagnóstico inicial	TGA no VSD	TGA with VSD
Padrão da artéria coronária no pré-operatório	Usual	Usual
Septostomia atrial com balão	Sim	Não
Peso (kg)	56,1	26,8
Altura (cm)	168,5	120,5
ECO	FEVE 72% Sem anormalidades da motilidade regional da parede ventricular	FEVE 73% Sem anormalidades da motilidade regional da parede ventricular
ECG	Sinus rhythm FC: 75 bpm	Sinus rhythm FC: 64 bpm
TCPE	TTR 1,1 Sem alterações no segmento ST	TTR 0,96 Sem alterações no segmento ST

*ECG: Eletrocardiografia, ECO: Ecocardiografia, FC: Frequência cardíaca, TGA: transposição das grandes artérias, DSV: defeito do septo ventricular, FEVE: fração de ejeção do ventrículo esquerdo, TTR: taxa de troca respiratória, TCPE: teste cardiopulmonar de exercício.*

## Discussão

A avaliação de pacientes submetidos à cirurgia de Jatene permanece um desafio clínico. Não existe consenso sobre o intervalo apropriado e o melhor método de imagem para monitoramento. Falta uma estratégia definitiva de abordagem quando anormalidades fisiológicas ou anatômicas subclínicas são identificadas, e sintomas atribuídos a potenciais complicações são raros. ^5^ Nenhum de nossos pacientes com anormalidades da artéria coronária apresentou achados anormais nos exames de rotina (ECG, ecocardiografia e TCPE), o que também foi descrito por outros autores. ^4^

A avaliação da circulação coronária após a cirurgia de Jatene permanece uma questão importante. Acotovelamento ( *kinking* ), estenose, e obstrução podem ocorrer em qualquer momento após a cirurgia de Jatene, e um padrão bimodal foi descrito. ^6^ Contudo, a real incidência de problemas tardios na artéria coronária após a cirurgia é desconhecida. Alguns estudos sobre estenose da artéria coronária relataram ausência de qualquer anormalidade, outros encontraram sua ocorrência em até 18% dos casos. ^9^ Em uma coorte de 130 crianças consecutivas com idade aproximada de cinco anos, Ou et al. relataram uma prevalência de 9,2% de lesões na artéria coronária. ^7^ No estudo de Tsuda et al., ^4^ dos 40 pacientes submetidos à angiografia coronária, 11 (27,5%) apresentavam anormalidades da artéria coronária. A maioria dos pacientes pareciam apresentar algum grau de espessamento intimal, mas a relevância clínica dessa informação ainda não foi esclarecida. ^10^ Apesar de o risco de isquemia miocárdica no período pós-operatório ter sido extensivamente descrito, o risco de lesões da artéria coronária e isquemia em longo prazo ainda não está claro. ^11^ Pacientes com lesões graves da artéria coronária podem não apresentar qualquer sintoma ou evidência de isquemia do miocárdio. ^3^ Uma vez que pacientes assintomáticos possam estar em risco, geralmente realiza-se investigação das artérias coronárias. A maioria das obstruções descritas são do óstio ou de tronco, causadas por compressão da porção inicial da artéria coronária. Portanto, avaliar a patência da artéria coronária no acompanhamento em longo prazo é essencial.

Contudo, um estudo de uma grande série mostrou que somente 0,26% dos pacientes que foram submetidos à cirurgia de Jatene passaram por alguma intervenção na artéria coronária no acompanhamento em longo prazo. ^12^ Ainda, uma meta-análise que incluiu 8798 pacientes em 66450 anos de acompanhamento mostrou que somente cinco mortes súbitas cardíacas ocorreram em pacientes assintomáticos. Em uma coorte de 647 pacientes acompanhados por 10 anos, só um paciente (0,1%) teve que ser reoperado por anormalidade da artéria coronária um ano depois da cirurgia de Jatene. ^11^

A maioria dos estudos sobre artérias coronárias no pós-operatório tardio de pacientes submetidos à cirurgia de Jatene foi realizado em pequenas amostras de pacientes que necessitaram de avaliação da artéria coronária por algum motivo. Apesar do pequeno número de pacientes incluídos no estudo, as artérias coronárias foram avaliadas em quase toda a coorte de pacientes. ^13^ De fato, 94% de nossos pacientes foram submetidos à avaliação multimodal. Não conseguimos identificar um fator de predisposição para obstrução coronária nesses pacientes. Alguns pacientes não conseguiram realizar o TCPE, e outros precisaram ser sedados antes da TCC. Não existe um consenso sobre quais pacientes devem ser submetidos à avaliação da artéria coronária após a cirurgia de Jatene. Enquanto vários grupos defendem que todo paciente submetido à cirurgia de Jatene deve ser avaliado no pós-operatório tardio, outros grupos afirmam que somente pacientes com anormalidades na avaliação clínica ou exames de rotina devem ser avaliados. Segundo estudos de séries de pacientes, ^6 , 14^ entre 27% e 100% dos pacientes operados são avaliados quanto à presença de doença da artéria coronária. Alguns autores recomendam uma avaliação angiográfica precoce em todos os pacientes, ^3^ apesar de uma meta-análise ter mostrado um risco de morte súbita cardíaca de somente 0,05%. ^11^ Outras séries apresentaram a necessidade de reintervenção coronária de somente 0,26% dos pacientes. ^12^

Em nossa coorte, foram incluídos pacientes com idade maior que cinco anos, sendo a mediana de 9,4 anos. Não existe consenso sobre o tempo apropriado de se investigar anormalidades da artéria coronária em pacientes submetidos à cirurgia de Jatene no pós-operatório tardio. Os pacientes são ou avaliados rotineiramente ou somente se apresentam achados anormais em exames de rotina como ETT e ECG. Os autores investigaram rotineiramente a circulação da artéria coronária em pacientes submetidos à cirurgia de Jatene três a oito anos após o procedimento. ^3 , 9^ Enquanto alguns autores relataram que não houve progressão das anormalidades da artéria coronária com o tempo, ^10^ outros afirmaram que a condição pode progredir. ^6^ Contudo, não há consenso se esses pacientes deveriam ser escaneados apenas uma vez ou a cada cinco anos após a cirurgia de Jatene. ^6 , 14^ Ainda, enquanto alguns autores sugerem uma angiografia coronária ao redor de 12 anos de idade, ^4^ outros sugerem que uma tomografia computadorizada (TC) pode ser realizada em todos os pacientes na puberdade. ^15^ Outros ainda recomendam que as artérias coronárias sejam rotineiramente examinadas após a idade de 17 anos. ^16^ Alguns centros recomendam a realização, na idade adulta, de TCC ou de angiografia coronária invasiva no mínimo uma vez em todos os pacientes que foram submetidos à cirurgia de Jatene. ^17^

Pacientes com estenose ou oclusão da artéria coronária podem ser assintomáticos, e achados ecocardiográficos podem ser elusivos. Anormalidades da motilidade regional da parede ventricular ou uma dilatação e disfunção ventricular progressiva podem sugerir estenose ou oclusão da artéria coronária, ^18^ mas esses achados são raros.

Os exames de rotina (ECG, ETT, TCPE) geralmente realizados no acompanhamento desses pacientes têm baixa sensibilidade (cerca de 43%), mas alta especificidade (cerca de 93%) para anormalidades da artéria coronária. ^6^ Alguns autores recomendam que pacientes submetidos à cirurgia de Jatene realizem um teste não invasivo para detectar isquemia a cada três a cinco anos. ^19^ Em nossa coorte, nenhum dos 61 pacientes apresentou resultado anormal no ECG ou ETT, e os dois pacientes com anormalidades coronárias também apresentaram TCPE normal. Apesar de o TCPE conseguir detectar possíveis sinais de isquemia em pacientes que se submeteram à cirurgia de Jatene, essas anormalidades não se correlacionam bem com estudos de perfusão. ^20 , 21^

Por muito tempo, a angiografia coronária seletiva foi considerada o método mais preciso para avaliação de obstrução da artéria coronária após a cirurgia de Jatene ^6 , 9^ principalmente pela sua grande disponibilidade. No entanto, o cateter coronário pode passar através de uma estenose ostial e falhar em indicar uma obstrução ostial. Além disso, as artérias coronárias não são visualizadas em estruturas adjacentes que possam comprimi-las ou dobrá-las. Ainda, a angiografia coronária seletiva é um procedimento invasivo com potencial risco vascular e requer exposição à radiação e anestesia geral em crianças. Mesmo depois que as artérias coronárias são avaliadas por TC, alguns grupos ainda realizam angiografia como um método de rotina, argumentando ser esse o método de escolha por permitir intervenção em caso de obstrução. ^3^

Na última década, no entanto, a TCC surgiu como um método mais seguro e mais rápido para avaliar a circulação da artéria coronária em comparação à angiografia coronária. A TCC de 64 cortes pode ser realizada com sucesso em crianças com idade maior que cinco anos e é sensível e específico para detectar estenose ou oclusão de artéria coronária após a cirurgia de Jatene. Exposição à radiação, uso de contraste iodado, e necessidade de acesso vascular são as desvantagens desse método. ^18^ A TC foi realizada em nossos pacientes de maneira segura, sem complicações.

Apesar de a ressonância magnética cardíaca conseguir avaliar artérias coronárias sem o uso de radiação ionizante, o método exige que o paciente permaneça quieto por 60 minutos, o que requer anestesia ou sedação em crianças com idade menor que nove anos. ^18^ A presença de aparelhos metálicos, como *stents* , pode interferir nas imagens. Uma vez que metade dos pacientes necessitariam de sedação, e seis outros pacientes com idade maior que seis anos apresentavam *stents* da artéria pulmonar, todos foram avaliados por TCC.

Nossos dois pacientes com anormalidades da artéria coronária não foram submetidos à nenhuma intervenção. Mesmo após o diagnóstico de uma oclusão ou obstrução, não existe consenso sobre a conduta, uma vez que a maioria dos pacientes são assintomáticos, sem alterações no ECG, ETT ou TCPE. Em várias séries, a taxa de reintervenção após o diagnóstico de alterações na artéria coronária variou de 3,8 a 12% dos pacientes investigados, ^6 , 7 , 9 , 14 , 15^ ou de 1 a 2% de todos os pacientes submetidos à cirurgia de Jatene. Ainda, existe normalmente um atraso entre o diagnóstico de lesão de artéria coronária e sua correção cirúrgica. Em uma série de pacientes com obstruções da artéria coronária, o tempo entre o diagnóstico de anormalidade da artéria coronária e sua correção foi, em média, de três anos, mas o intervalo entre o diagnóstico e o tratamento cirúrgico poderia chegar a oito anos. ^14 , 15^ Os pacientes foram submetidos a alargamento cirúrgico do óstio coronário, enxerto da artéria mamária, ou dilatação com balão e implante de *stent* . ^7 , 14 , 15^

Uma revisão sistemática que analisou 8798 pacientes mostrou que somente cinco (0,05%) apresentaram morte cardíaca súbita por anormalidades da artéria coronária, apesar que 7,3% dos pacientes apresentaram algum grau de obstrução coronária. Outro ponto a considerar é que 4,9% dos pacientes que tiveram que se submeter a reintervenção por obstrução da artéria coronária morreram. Em outra série de 7951 pacientes, somente 0,26% necessitaram de intervenção da artéria coronária, e a taxa de mortalidade foi de 20%. ^12^

O presente estudo incluiu uma coorte de pacientes de uma única instituição, de modo que viés de seleção pode ter ocorrido. O número de casos também pode ter sido inadequado para mostrar uma correlação entre o diagnóstico inicial ou o padrão inicial da artéria coronária e subsequente anormalidades.

## Conclusões

Em nosso estudo, a incidência de anormalidades da artéria coronária após a cirurgia de Jatene foi de 3,3%, e todos eram assintomáticos. Apesar de a comunidade médica ter que estar atenta a tais condições após a cirurgia de Jatene, não existem diretrizes claras sobre quando e como essas artérias devem ser abordadas e sobre o que fazer se as alterações na artéria coronária ocorrerem em pacientes assintomáticos.

Nosso estudo mostrou que os pacientes com obstrução anatômica nas artérias coronárias podem ser assintomáticos mesmo ao TCPE, e com ECG e ETT normais. Uma abordagem multimodal, com informação funcional e anatômica é ainda necessária. Com base nos nossos achados, decidimos manter um seguimento anual com ETT, ECG e TCPE. Se os pacientes apresentam sintomas clínicos (arritmia, dor torácica, ou fadiga excessiva), ou uma alteração em um exame de rotina, recomendamos uma TCC.
