# Supplementary material for: Avaliação da Circulação Coronariana após a Cirurgia de Jatene
Source: Arq Bras Cardiol. 2021 Jun 8;116(6):1111–6. [Article in Portuguese] doi: 10.36660/abc.20200095 (PMC8288536; doi:10.36660/abc.20200095)
Supplement: Translation

# Translation: Evaluation of Coronary Circulation after Arterial Switch Operation

**Authors:** Mariana Nicoletti Ferreira Baldo, Henrique Simão Trad, Tarcisio José da Silva, Paulo Henrique Manso

**Affiliations:** 1 Brasil Universidade de São Paulo, Ribeirão Preto, SP - Brasil; 2 Brasil Lotus Radiologia, Ribeirão Preto, SP – Brasil

**Keywords:** Coronary Artery, Coronary Circulation, Jatene’s Surgery, Arterial Switch Operation, Tomography, X-Ray, Diagnostic, Imaging

## Abstract

**Background:**

Coronary artery evaluation remains after arterial switch operation a clinical challenge.

**Objective:**

This study aims to correlate anatomical changes diagnosed by cardiac computed tomography (CCT) with physiological alterations on clinical evaluation to diagnose coronary obstruction in late ASO patients.

**Methods:**

This study included 61 consecutive patients with mean age of 9.4 years who underwent ASO. The patients were submitted to echocardiography, electrocardiography, cardiopulmonary exercise test, and cardiac computed tomography to evaluate functional capacity and coronary artery anatomy.

**Results:**

Cardiac computed tomography revealed that only 3.3% of the patients had coronary stenosis. These patients were asymptomatic, and no signs of myocardial ischemia were detected by the tests.

**Conclusion:**

The incidence of coronary abnormalities in late ASO patients was 3.3% in our cohort. There is no clear guideline as to why, when, and how these patients should be screened or what to propose when a coronary obstruction is diagnosed in asymptomatic patients.

## Introduction

Although arterial switch operation (ASO) is associated with a low early mortality and morbidity in most centers,^1^ late complications, such as obstructive coronary artery lesions, right ventricular outflow tract obstruction, and neoaortic root dilation and regurgitation may be present in up to 25% of the cases.^2^ In addition, late abnormalities in coronary artery circulation have been reported to occur in up to 18% of the cases in late follow-up.^3^ However, the true incidence of late coronary artery problems after ASO is unknown because most patients with coronary artery stenosis or occlusion may be asymptomatic, the reported incidences tend to depend on the depth of investigations.^4^ Besides, there are no clear guidelines about when these patients should be screened or the best screening method in this situation.^5^

In addition to clinical evaluation, ASO patients require a multimodality assessment for possible late complications. Transthoracic echocardiography (TTE), electrocardiography (ECG), and cardiopulmonary exercise test (CPET) are usually used for the long-term follow-up of these patients. Nevertheless, these screening methods are not sensitive enough to detect coronary artery abnormalities.^6^

Cardiac computed tomography (CCT) is a good method to evaluate coronary artery anatomy of ASO patients in the late post-operative phase, with a high spatial resolution within a short acquisition time. CCT is an ideal method for patients that need detailed evaluation of reimplanted coronary arteries.^7^

## Objective

To evaluate coronary artery circulation in a cohort of ASO patients in the late operative period by TTE, ECG, CPET, and CCT.

## Methods

This prospective study was approved by the local institutional review board (43493315.6.0000.5440). Patients that were submitted to ASO at our institution between 1998 and 2009 were recruited. Inclusion criteria were age over five years, and written consent of the parents to participation. Patients with known allergy to iodinated contrast were excluded from the study. Medical data regarding initial diagnosis, coronary artery anatomy, and age at surgery were collected from the medical records. The patients were then submitted to ECHO, ECG, CCT, and CPET within a four-month period.

ECG and TTE were performed according to routine protocols. CPET was performed on an inclined treadmill at constant speed, with an increase in the inclination during the test.

CCT was performed in a 64-slice device (Somaton Sensation, Siemens, Germany). When necessary, an oral beta-blocker was administered two hours before the exam so that a heart rate below 80 bpm would be achieved. The participating children had been previously trained to hold their breath for 10 seconds. When the patient was not able to cooperate, intravenous midazolam was given (0.1 to 0.2 mg/kg). Image acquisition parameters were adapted to enable the use of the lowest radiation dose possible. Two independent radiologists analyzed the images.

### Statistical analysis

A descriptive analysis of the data was carried out. Continuous data are expressed as median and range; categorical data are expressed as percentage. All statistical analyses were performed using GraphPad Prism 5.0 (GraphPad Software, La Jolla, CA, USA) with the level of statistical significance set at P< 0.05.

## Results

### Patient demographics

Of the 69 initially recruited patients, four did not agree to participate, two missed follow-up, and two died (one patient died during pulmonary artery stenting in the cath lab, and one patient died of unknown cause). The remaining 61 patients were submitted to ECG and TTE; 60 patients were subjected to CCT; and 51 patients did CPET. All patients were asymptomatic and were not using any cardiac medications.

Seventy percent of the patients were male. Most patients had transposition of the great arteries (TGA) with intact interventricular septum (56.7%), 31.7% had TGA with ventricular septal defect (VSD), and 11% had the Taussig-Bing anomaly. In 90% of the patients, the coronary artery arose from its usual origin in the pre-operative period (the left coronary arose from sinus 1 and right coronary arising from sinus 2).

ASO was performed at a mean of 14 days (range: 2 to 38 days), median age of participants was 9.4 years (5 to 18 years), median weight was 29.9 Kg (20 to 84 Kg), and median height was 134 cm (112 to 183 cm).

### Clinical investigation

All patients had ECG and TTE results. Most patients (96%) were in sinus rhythm, while 4% had right atrial rhythm. No patient had ST changes or ventricular ectopy in the resting ECG. The TTE showed that all patients had normal ejection fraction (>55%) and normal ventricular regional wall motion.

CPET: Fifty-one patients were able to perform the CPT (10 patients had limb or neurological conditions and were not able to perform the test), and none of them presented ST segment abnormalities or arrhythmia during the test. They presented a VO_2_max of 31.7 mL/Kg/min (22.3 – 43.2) and reached a mean of 9 (6.4-12.3) METS.

CCT: Sixty patients were able to undergo CCT. The mean dose length product (DLP) was 138 (56 -490) mGy-cm; the mean dose was 2 (0.9–8.7) mSv. Only two (3.3%) patients had coronary artery abnormalities; one of the patients had moderate left coronary artery stenosis, and the other presented severe right coronary artery obstruction ( Figure 1 ). We graded coronary artery obstruction according to published guidelines.^8^ Both patients were asymptomatic, and all the other tests (resting ECG, TTE, and CPET) revealed normal findings ( Table 1 ). We could not find any correlation between primary cardiac diagnosis or coronary artery pattern and the presence of coronary stenosis.

**Figure 1:** – Cardiac computed tomography image; right coronary artery with severe obstruction (arrow)

**Table 1:** – Characteristics of the patients with coronary artery abnormalities as demonstrated by cardiac CT

	Patient 1	Patient 2
Coronary artery lesion	Moderate left coronary artery occlusion	Severe right coronary artery obstruction
Age (years)	14.7	6.6
Gender	Female	Male
Initial diagnosis	TGA without VSD	TGA with VSD
Pre-operative coronary artery pattern	Usual	Usual
Balloon atrial septostomy	Yes	No
Weight (kg)	56.1	26.8
Height (cm)	168.5	120.5
ECHO	LVEF 72% No regional wall motion abnormalities	LVEF 73% No regional wall motion abnormalities
ECG	Sinus rhythm HR: 75 bpm	Sinus rhythm HR: 64 bpm
CPET	RER 1.1 No ST-segment changes	RER 0.96 No ST-segment changes

*ECG: Eletrocardiography; ECHO: Echocardiography; HR: Heart Rate; TGA: transposition of the great arteries; VSD: Ventricular Septal Defect; LVEF: Left Ventricle Ejection Fraction; RER: Respiratory Exchange Rate; CPET: Cardiopulmonary Exercise Test.*

## Discussion

Evaluation of patients undergoing ASO remains a clinical challenge: there is no consensus regarding the appropriate interval and modality for surveillance imaging. A defined management strategy is lacking when subclinical anatomical or physiological abnormalities are identified; and symptoms attributable to potential complications are rare.^5^ None of our patients with coronary artery abnormalities had abnormal findings in routine tests (ECG, echocardiography, and CPET), which has also been described by other authors.^4^

Evaluation of the coronary circulation after ASO remains an important issue. Kinkings, stenosis, and obstruction may arise any time after ASO, and a bimodal pattern has been described.^6^ However, the true incidence of late coronary artery problems after ASO is unknown. While some studies on coronary artery stenosis have reported no abnormalities at all, other studies have reported the occurrence in up to 18% of the cases.^9^ In a cohort of 130 consecutive children aged about five years, Ou and colleagues showed a prevalence of 9.2% of coronary artery lesions.^7^ In the study by Tsuda et al.,^4^ of 40 patients submitted to coronary angiography, 11 (27.5%) had coronary artery abnormalities. Most patients seemed to have some degree of intimal thickening, but the clinical relevance of this information still has not been understood.^10^ Although the risk of myocardial ischemia in the postoperative period has been extensively described, the long-term risk for coronary artery lesions and ischemia remains unclear.^11^ Patients with severe coronary artery lesions may not show any symptoms or evidence of myocardial ischemia.^3^ Because asymptomatic patients might be at risk, coronary arteries are often investigated. Most of the described obstructions are ostial or stem obstruction, caused by compression in the initial portion coronary artery. Therefore, investigating coronary artery patency in the long-term follow-up is essential.

Nevertheless, a paper on a large series showed that as few as 0.26% of patients who had undergone ASO were submitted to coronary artery interventions in the long-term follow-up.^12^ Moreover, a meta-analysis that enrolled 8,798 patients in 66,450 patient follow-up years showed that only five sudden cardiac deaths occurred in asymptomatic patients. In a cohort of 647 patients followed up for 10 years, only one patient (0.1%) had to be re-operated due to coronary artery abnormality one year after ASO.^11^

Most studies regarding coronary arteries in the late post-operative period after ASO have been conducted in small samples of patients that required coronary artery evaluation for some reason. Despite the small number of patients included in the present study, the coronary arteries were evaluated in almost our entire cohort of patients.^13^ Indeed, 94% of our patients underwent multimodal assessment. We could not identify a predisposition factor for coronary obstruction in these patients. Some patients were not able to perform the CPET, and other patients had to be sedated before CCT. There is no consensus about who should be assessed for coronary artery disease following ASO. While several groups advocate that every ASO patient should be investigated in the post-operative period, other groups claim that only patients with abnormalities in clinical examination or routine tests should be evaluated. Series have varied from 27% to 100% of ASO patients undergoing assessment for coronary artery disease.^6 , 14^ Some authors recommend that an early angiographic evaluation be conducted in all patients^3^ even though a meta-analysis has shown a sudden cardiac death chance of only 0.05%.^11^ Other series have shown the need for coronary reintervention in only 0.26% of the patients.^12^

In our cohort, patients older than five years were enrolled; the median age was 9.4 years. There is no consensus concerning the appropriate time to look for coronary artery abnormalities in ASO patients in the late operative period. Patients are either routinely investigated or are only investigated when they present any clinical sign or abnormal findings in routine exams like TTE and ECG. Authors have investigated coronary artery circulation routinely in ASO patients three to eight years after the surgery.^3 , 9^ Some authors found that coronary artery abnormalities did not progress over time,^10^ while others noted progression that the coronary artery abnormalities may be progressive.^6^ However, there is no consensus as to whether these patients should be screened once in a lifetime or every five years after ASO.^6 , 14^ Also, whenever some authors suggest a coronary angiogram around 12 years of age,^4^ others suggest that a computed tomography (CT) scan be performed in all patients at puberty.^15^ Other authors recommend that coronary arteries be routinely investigated after 17 years of age.^16^ Some centers recommend CCT or invasive coronary angiography at least once in adulthood in all ASO patients.^17^

Patients with coronary artery stenosis or occlusion may be asymptomatic, and echocardiography findings may be elusive. Regional wall motion abnormalities or progressive ventricular dilation and dysfunction may arouse suspicion of coronary artery stenosis or occlusion,^18^ but these findings are rare.

Although the routine tests (ECG, TTE, CPET) usually performed in the follow-up of these patients provide low sensitivity (about 43%), but high specificity (about 93%) for coronary artery abnormalities.^6^ Some authors recommend that adult ASO patients perform a noninvasive ischemia testing every three to five years.^19^ In our cohort, none of the 61 patients had any abnormal finding in the ECG or TTE, and the two patients with coronary abnormalities also had normal CPET. Although the CPET may detect signs of ischemia in patients after ASO, these abnormalities do not correlate well with perfusion studies.^20 , 21^

For a long time, selective coronary angiography was considered the most accurate method to assess coronary artery obstruction after ASO^6 , 9^ mostly because of its wide availability. Nevertheless, the coronary catheter may pass through an ostial stenosis and fail to visualize an ostial obstruction. In addition, the coronary arteries are not visualized in the context of adjacent structures that may compress or kink said arteries. Furthermore, selective coronary angiography is an invasive procedure with potential vascular risk and requires exposure to radiation and general anesthesia in young children. Even after the coronary arteries are evaluated by CT, some groups still perform angiography as a routine method, considering it as the method of choice since it allows intervention if an obstruction is diagnosed.^3^

In the last decade, however, CCT has emerged as a safer and faster method to evaluate coronary artery circulation as compared to coronary angiography. Sixty-four-slice CCT can be successfully performed in children aged over five years and is both sensitive and specific to detect coronary artery stenosis or occlusion after ASO. Exposure to radiation, use of iodinated contrast, and need for a vascular access are the drawbacks of this method.^18^ CT was performed in our patients safely, without complications.

Although cardiac magnetic resonance can assess coronary arteries without the use of ionizing radiation, it requires that the patient remain still for 60 minutes, and thus also anesthesia or sedation in children aged less than nine years.^18^ The presence of metallic devices, like stents, may interfere in the images. Since half of our patients would need sedation, and six other patients older than nine years had pulmonary artery stents, all of them were evaluated by CCT.

Our two patients with coronary artery abnormalities were not submitted to any intervention. Even after an occlusion or obstruction is diagnosed, there is no consensus as to what should be done because most of the patients are asymptomatic without alterations in ECG, TTE, or CPET. In several series, the rate of re-intervention after diagnosis of coronary artery alterations varied from 3.8 to 12% of the investigated patients^6 , 7 , 9 , 14 , 15^ or 1 to 2% of all patients undergoing ASO. Moreover, there is usually a delay between the coronary artery lesion diagnosis and its surgical correction. In one series of patients with coronary artery obstructions, the time elapsed between the diagnosis of coronary artery abnormality and its repair was three years on average, but the gap between diagnosis and surgical treatment could be as long as eight years.^14 , 15^ Patients were submitted to surgical coronary ostium enlargement, mammary artery graft, or balloon dilation and stenting.^7 , 14 , 15^

A systematic review that analyzed 8,798 patients showed that only five patients (0.05%) had sudden cardiac death due to coronary artery abnormalities, although 7.3% of patients presented some level of coronary obstruction. Another issue to consider is that 4.9% of the patients that had to undergo re-intervention due to coronary artery obstruction died. In another large series of 7,951 patients, only 0.26% needed coronary artery intervention and the mortality rate was 20%.^12^

The present study involved a single-institutional patient cohort, so selection bias may have occurred. The number of cases may also be inadequate to show a correlation between initial diagnosis or original coronary artery pattern and subsequent coronary abnormalities.

## Conclusions

In our study, the incidence of coronary artery abnormalities after ASO was 3.3%, and all of them were asymptomatic. Although the medical group must be aware of these conditions after ASO, there are no clear guidelines as to when and how these coronaries should be addressed or about what to do if coronary artery alterations arise in asymptomatic patients.

Our study has shown that patients with anatomical obstruction in coronary arteries may be asymptomatic even at CPET, and with normal ECG and TTE. A multimodality approach with functional and anatomical information is still necessary. Based on our findings, we have decided to keep an annual follow-up with TTE, ECG, and CPET. If the patient presents clinical symptoms (arrhythmia, thoracic pain, or excessive fatigue) or an alteration in a routine exam, we recommend a CCT.
